# Attitudes Toward a Proposed GPS-Based Location Tracking Smartphone App for Improving Engagement in HIV Care Among Pregnant and Postpartum Women in South Africa: Focus Group and Interview Study

**DOI:** 10.2196/19243

**Published:** 2021-02-08

**Authors:** Kate Clouse, Tamsin K Phillips, Phepo Mogoba, Linda Ndlovu, Jean Bassett, Landon Myer

**Affiliations:** 1 Vanderbilt University School of Nursing Nashville, TN United States; 2 Vanderbilt Institute for Global Health Vanderbilt University Medical Center Nashville, TN United States; 3 Division of Epidemiology and Biostatistics School of Public Health and Family Medicine University of Cape Town Cape Town South Africa; 4 Witkoppen Clinic Johannesburg South Africa

**Keywords:** HIV/AIDS, South Africa, smartphone, mobile health, pregnancy, GPS tracking

## Abstract

**Background:**

Peripartum women living with HIV in South Africa are at high risk of dropping out of care and are also a particularly mobile population, which may impact their engagement in HIV care. With the rise in mobile phone use worldwide, there is an opportunity to use smartphones and GPS location software to characterize mobility in real time.

**Objective:**

The aim of this study was to propose a smartphone app that could collect individual GPS locations to improve engagement in HIV care and to assess potential users’ attitudes toward the proposed app.

**Methods:**

We conducted 50 in-depth interviews (IDIs) with pregnant women living with HIV in Cape Town and Johannesburg, South Africa, and 6 focus group discussions (FGDs) with 27 postpartum women living with HIV in Cape Town. Through an open-ended question in the IDIs, we categorized “positive,” “neutral,” or “negative” reactions to the proposed app and identified key quotations. For the FGD data, we grouped the text into themes, then analyzed it for patterns, concepts, and associations and selected illustrative quotations.

**Results:**

In the IDIs, the majority of participants (76%, 38/50) responded favorably to the proposed app. Favorable comments were related to the convenience of facilitated continued care, a sense of helpfulness on the part of the researchers and facilities, and the difficulties of trying to maintain care while traveling. Among the 4/50 participants (8%) who responded negatively, their comments were primarily related to the individual’s responsibility for their own health care. The FGDs revealed four themes: facilitating connection to care, informed choice, disclosure (intentional or unintentional), and trust in researchers.

**Conclusions:**

Women living with HIV were overwhelmingly positive about the idea of a GPS-based smartphone app to improve engagement in HIV care. Participants reported that they would welcome a tool to facilitate connection to care when traveling and expressed trust in researchers and health care facilities. Within the context of the rapid increase of smartphone use in South Africa, these early results warrant further exploration and critical evaluation following real-world experience with the app.

## Introduction

The World Health Organization endorsed universal, lifelong antiretroviral therapy (ART) for all pregnant and breastfeeding women in 2013 [1], followed in 2015 by universal ART for all adults and children with HIV [2]. Expansion of HIV treatment delivers undeniable health benefits, including substantial gains in life expectancy [3]; however, challenges arise in ensuring continuous, lifelong engagement in HIV care [4]. Peripartum women living with HIV are at especially high risk of dropping out of care, particularly after delivery [5,6]. South Africa is home to the world’s largest ART program, and the country adopted universal ART in 2016; however, studies have found suboptimal engagement in peripartum HIV care [7-9].

Population mobility affects engagement in HIV care as a barrier to both individual access to health care facilities and the ability to determine if an individual is truly lost from care. Individuals who drop out of care at one facility may continue at a second facility, known as “silent” or “unofficial” transfer [10]; this may underestimate engagement [11], given the absence of linked data networks at health care facilities [12]. Long-distance travel or relocation may also result in disengagement from HIV care or treatment disruption if an individual is unable to locate a new facility.

The population of South Africa is highly mobile both internally (circular migration) and across international borders [13]. Peripartum women are a particularly mobile population, with one study finding that nearly half of pregnant and postpartum women traveled during the peripartum period—especially after delivery—to destinations including eight of South Africa’s nine provinces and four foreign countries [14]. To understand the impact of mobility on engagement in HIV care, better measurement tools must be implemented to accurately track individuals’ movements [15]. With the rise in mobile phone use worldwide, there is an opportunity to use smartphones and GPS location software to characterize mobility in real time.

Previously, researchers have demonstrated the use of bulk cell phone data to model infectious disease spread due to mobility [16], internal mobility within a country [17], and population movement after a major natural disaster [18]. These studies were conducted using proprietary network data from commercial cell phone service providers; however, this requires the cooperation of each provider. Also, use of aggregate cell phone service data requires intense computational power, does not allow for individual consent, and may be subject to breaches of security and misinterpretation of data [15].

In South Africa, smartphone ownership has seen tremendous growth [19]; concurrently, mobile health (mHealth) interventions have demonstrated acceptability among local populations [20-22]. This context provides an ideal opportunity to explore population mobility and intervene in health outcomes at an individual level, given that individuals typically carry their mobile phones with them all day [23]. Although the use of GPS to track individual movement is a common feature of commercial smartphone apps, it has not been widely reported in the context of mHealth. However, two New York City–based studies of wearable GPS devices for the purpose of tracking daily mobility were found to be acceptable among people living with HIV [24,25].

Given the rising use of smartphones and the urgent need to better understand population mobility and its impact on engagement in HIV care, we set out to develop a smartphone app, CareConekta, with two primary functions. Firstly, the app will prospectively collect consenting individuals’ GPS “fuzzy” location data (within one kilometer of their actual location) to better understand patient mobility; importantly, it will also enable real-time intervention to improve engagement when an individual has traveled away from their primary facility. Parameters can be set, such as a time or distance from the clinic area, to initiate contact and assist with facility transfer and medication refills. Secondly, the app will contain nationwide facility information to serve as a “clinic finder” when seeking a new facility. Initially, the app will be aimed at pregnant and postpartum women, a population known to be mobile and at high risk of disengagement from HIV care. The results presented here are part of the formative research undertaken prior to developing this app, assessing attitudes toward this concept among pregnant and postpartum women living with HIV in South Africa.

## Methods

We used two approaches (detailed below) to collect data for this analysis.

### In-depth Interviews

We conducted semistructured in-depth interviews (IDIs) with 50 adult (aged ≥18 years) pregnant women living with HIV recruited during routine antenatal care at two primary care facilities in Johannesburg and Cape Town, South Africa. Interviews were conducted from September 2016 to September 2017 by an experienced qualitative researcher based at each site in the preferred local language of the participant and were recorded, transcribed, and translated into English as previously reported [26]. The primary objective of the IDIs was to characterize and understand motivations for mobility during the peripartum period; however, we also asked a series of open-ended questions to assess respondents’ attitudes toward proposed interventions to improve engagement in HIV care among peripartum women. Of these open-ended questions, only one related to a proposed location-tracking intervention:

Some cell phone programs can track where a person travels. For example, a program could use the signal from someone’s cell phone to alert the clinic to when that person was out of town, so that the clinic could check with her about her supply of tablets or upcoming visits. What do you think about this? Is this something that you would be interested in having on your phone?

Due to the limited nature of this question, we did not take a typical qualitative approach to this part of the analysis but instead quantitatively assessed the proportion of participants responding favorably or unfavorably. Two reviewers (KC and TP) independently assessed each response and rated it as “favorable,” “negative,” or “neutral” based on whether the participant would be interested in having the proposed intervention on their own phone. We then assessed the interrater reliability and reached agreement on discordant findings.

### Focus Group Discussions

We also conducted focus group discussions (FGDs) at the same primary care facility in Cape Town where the IDIs were conducted, using a private room that accommodated seating for all participants. The objectives of the FGDs were to explore mobile phone use and preferences among postpartum women living with HIV in South Africa and to engage potential users to identify core design elements promoting the usability and acceptability of a mobile phone app. Focus group discussions were selected as the data collection method to enable group interaction and exchange of ideas as well as to foster deeper discussion than is possible with the open-ended questions in the IDI. The recruitment and enrollment information are described in greater detail elsewhere [27]. From January to March 2017, we enrolled 27 adult (≥18 years), recently postpartum (<12 months since delivery) women living with HIV who currently used smartphones and attended regular care at the study site in the 6 FGDs. The participants were asked to pick a color to use instead of their names during the discussion. The FGDs were moderated by a local research coordinator, conducted in isiXhosa (the predominant local language), recorded, transcribed, and translated to English. Approximately half of the FGD guide focused on questions related to the proposed CareConekta app (Multimedia Appendix 1).

### Data Analysis

Quantitative data were analyzed using SAS 9.4 (SAS, Inc), reporting proportions for categorical data and medians and IQRs for continuous variables. The transcripts of the FGDs were analyzed in NVivo 11 (QSR International). We took a deductive approach to framework analysis by identifying codes using research questions [28]. We focused on 3 topics, guided by the interview questions: attitudes toward a clinic finder feature, attitudes toward a mobility tracking feature, and privacy concerns. Data were analyzed by grouping the text into themes. The text under each theme was then examined for convergent and divergent patterns, concepts, and associations. Illustrative quotes were selected to elucidate the findings.

### Ethical Review Statement

All participants provided written informed consent prior to the study activities. The research activities were approved by the ethical review boards of Vanderbilt University, the University of the Witwatersrand, and the University of Cape Town.

## Results

### Participant Characteristics

The 50 IDI participants had a median age of 29.5 years (IQR 24-34) and a median duration on ART of 9.4 months (IQR: 2.0-38.6). The median age of the 27 FGD participants was 30 years (IQR 23-34), the median time since delivery was 6.5 months (IQR 2.4-9.4), and the median duration on ART was 16.1 months (IQR: 10.6-51.2).

### IDIs

Of the 50 participants, 76% (38/50) responded positively to the proposed tracking intervention, 16% (8/50) were neutral, and 8% (4/50) responded negatively. Among those who responded favorably, their comments related to the convenience of facilitated continued care, a sense of benevolence on the part of the researchers and/or health care facility for providing this service, and the difficulties of trying to maintain care while traveling:

It would show me that I am cared for, and that would encourage me.

Participant 15, favorable

I think that would be fine because people sometimes travel unexpectedly and have not come to the clinic to get medication.

Participant 17, favorable

Maybe when I arrive at [village name], I might not adhere to my treatment. This will still help me, you understand. I might be followed up by the health care workers of the place, because they will be in possession of my details.

Participant 18, favorable

I would love that, because it would remind me.

Participant 33, favorable

Among the minority of participants who responded negatively and said they would not want the app, or who were neutral and said they were ambivalent about the app, the comments primarily related to the individual’s responsibility for their own health care rather than the facility’s:

I think the patient should be the one who is active about such a thing.

Participant 42, neutral

I have been out of town ever since I started my treatment. What would help me [more] is to come to the clinic to ask for transfer letter because it [the clinic] might not know if I will spend some time in Eastern Cape [Province].

Participant 9, neutral

That is somehow an invasion of privacy…I mean, we’re all adults. If you have to travel and you know your status, you just need to make sure that you’ve got enough medication for you to be able to travel.

Participant 29, negative

If a person is serious about life, they will ask for a transfer letter.

Participant 40, negative

A small group of participants misunderstood the concept of tracking to mean that researcher would be able to see them through their phone, responding that researchers should not be able to “see things that you are not supposed to see” [Participant 41], such as when the participant is naked.

### FGDs

Four key themes emerged during the FGDs: facilitating connection to care, informed choice, disclosure (intentional or unintentional), and trust in researchers.

#### Attitudes Toward the Clinic Finder Feature

Respondents were overwhelmingly positive toward an app that would offer clinic location information, as the improved ability to connect to care was perceived as a major benefit. Responses related to this theme included:

For example, I am going to Eastern Cape [Province]…Once I run out of my treatment, my pills, even if I don’t have money, I would have to rush—even if I travel by debt—back to Western Cape again because I used to come to the clinic here. So, it would make my life on the other side to be so easy.

Yellow, FGD1

One respondent noted the benefit of linking to care for her child:

I think it is the right thing because I travelled with [my] boy to Durban; he was one month old. So when I got to Durban, there is no clinic I knew of…I was forced to come back to Cape Town because my baby had to get his injections. Do you see that if there was an app I would have been able to look which is the nearest clinic around. I think it would help very much.

Pink, FGD5

Similarly, respondents reported that a clinic finder feature would enable them to make a more informed choice by providing more information about clinic services and location: “Clinics are not the same; there are children’s clinics and adults’ clinics as well, so before I go I should know first if it is the right clinic” [White, FGD5]. One response highlighted issues of both continuity of care and informed choice, noting a clinic finder feature would allow her, when traveling, to “go with confidence so you know how to approach the nurses, because your app assured you that these services are available” [Maroon, FGD6]. Interestingly, respondents expressed a desire to use a clinic finder feature locally to find new clinics offering privacy and anonymity from community members who may not know their HIV-positive status. For example:

I think it would help very much because sometimes…your [HIV] status is unknown and you are not ready to tell in the area that you are in that I am this kind of person [HIV-positive], so it would be better to search for yourself and go without asking anyone.

Green, FGD3

In FGD1, two participants misunderstood the proposed feature, thinking that they would be required to go to the closest clinic identified by the proposed app. They noted that they wished to avoid this because “most of the people who are working at the [closest clinic], they are from my village, so I am afraid” [Gold, FGD1]. These responses underscore the issue of the desire to choose one’s clinic to avoid disclosure of disease status.

#### Attitudes Toward the Mobility Tracking Feature

Overall, the respondents were supportive of the idea of allowing researchers to track their location. Positive responses noted the usefulness of this idea for connecting to care in the event of missed clinic visits, informing the home clinic about the participant’s current location, and providing potential for information to be shared back to the participant:

I think it is good that they know your location, for maybe you have missed the [visit] date.

Red, FGD2

I think it is right to record [location] so that they are able to get [connect] you.

White, FGD2

You would be in East London, perhaps, in the meantime it [clinic] would still assume that you are here in Cape Town, so I think it is right about recording [location] because it would know that you are in East London now, so it should search for nearby ART clinics there.

Red, FGD5

Sharing of location information with researchers was noted as acceptable, suggesting that the participants find it easier to discuss HIV with researchers than with people in their family or community to whom they may not wish to disclose their HIV status:

I would like that. I just would not like to share information with my neighbors. Researchers from UCT [University of Cape Town] do not know me anyway.

Black, FGD6

I also would love to be located by UCT, not people who know me.

White, FGD6

A few participants misunderstood the concept of a mobility tracking feature, confusing the concept of “recording” location with “recording” a message: “Something that is recorded stays, so you will be able to listen again or to call it again” [Yellow, FGD2]. Additionally, a few participants believed that a mobility tracking feature could function as a “find my phone” device, which is outside of the scope of this feature.

#### Privacy Concerns

When asked about privacy-related issues related to a clinic finder or mobility tracking feature, few concerns were noted. Most privacy concerns related to the clinic finder feature and receiving messages informing participants of nearby facilities rather than to the location tracking feature. Respondents indicated that prior disclosure of their HIV-positive status to friends and family members meant that they had few concerns with unintentional disclosure via their mobile devices. Occasional phone sharing with sisters, mothers, and partners was noted, but unintentional disclosure to these individuals was not raised as a concern:

There is nothing that I am hiding.

Black, FGD2

I have no concerns because my phone doesn’t stay with [is not shared with] many people; it stays with people who know my status.

Red, FGD5

However, one group raised fears of more general unintentional disclosure, possibly misunderstanding the tracking feature: “In this app, names of [HIV-] positive people are going to be recorded” [White, FGD2].

Overall, however, this was a minority perspective, and the idea was raised that the researchers were “helping” participants by offering these app features. An understanding of the confidentiality of research was noted by statements such as “I am not worried at all because my name is not used in the study. It remains confidential” [Black, FGD6]. Trust in the researchers was also noted, with one participant stating, “I don’t see any issues because these people [researchers and health care providers] are helping us” [Maroon, FGD6].

## Discussion

Through our research with pregnant and postpartum women living with HIV in South Africa, we found high potential acceptability for a proposed app that would use GPS location information to track mobility and provide a clinic finder function. In recent years, the high prevalence of cell phones has spurred the creation of numerous mHealth interventions [29,30]. South Africa has been a leader in the development and widescale implementation of mHealth interventions in sub-Saharan Africa, with the notable example of MomConnect, a text message–based health information program for peripartum women that reached >60% coverage of pregnant women attending antenatal care nationally [31]. With the increased availability of more sophisticated mobile devices, such as smartphones, potential has emerged to access individual GPS location information to improve health outcomes. Our work developing the novel CareConekta app represents an early example of using GPS technology and individual location tracking within the mHealth context.

From our earliest discussions about developing a smartphone app to collect individual location data, privacy has been at the forefront of our concerns. Our motivation for this work was to understand the attitudes of potential users—pregnant and postpartum women living with HIV—toward such an intervention as well as their privacy concerns prior to developing the app. In discussing privacy, we were surprised that respondents mentioned using a GPS-based clinic finder locally—in the absence of travel—to find a new facility that promised anonymity and security from unintentional disclosure. These remarks underscore the continued heavy burden of HIV stigma within the context of a generalized epidemic and universal testing and treatment [32]. Our findings also suggest a high level of trust between respondents and researchers, demonstrating an understanding of confidentiality practices within research and expressing a sense of helpfulness on the part of the researchers. Although few privacy concerns were raised, issues of stigma and individual privacy concerns could be addressed through an app design that is not specific to HIV. As previously reported, approximately half of the FGD participants shared their phones, primarily with family members and friends [27]. However, most of the participants suggested that this sharing was short-term, such as allowing a friend to check Facebook. Further research is warranted to investigate the feasibility of mHealth interventions specific to individuals, such as those tracking GPS location, in the context of potential phone sharing.

In both the IDIs and the FGDs, some misunderstandings of the proposed app and its features were expressed. These ranged from fearing that researchers would be allowed to see participants through the smartphone to incorrect assumptions of benefits, such as using the app to find one’s misplaced phone. This emphasizes the need for substantial training of the staff responsible for implementation; also, a thorough introduction to the app will be needed for participants during enrollment, which must include the potential limitations of the app and also highlight these concepts.

Strengths of our study include representing participant responses at two geographic sites in South Africa and using two distinct methods for collecting extensive participant feedback: IDIs and FGDs. Study limitations include that the IDIs and FGDs were not designed to detect differences across study sites or to enable participant subgroup analyses. Participation in the FGDs was limited to women currently using smartphones attending a single clinic in Cape Town. Our sample size was small compared to those of quantitative studies but was appropriate for qualitative research and enabled meaningful discussion. Data were collected from September 2016 to September 2017 and represent the respondents’ attitudes at that specific time. At both study clinics, the participants were aware of health research; therefore, research in different populations and settings is needed to determine whether the low privacy concerns and high trust in researchers reported are generalizable. The attitudes reported here are based on a hypothetical app that was only briefly introduced and not viewed or used by the participants. This required the participants to think abstractly about the proposed app and may have produced biased results that will be revealed when an actual app is developed and tested. Thus, it is important to follow this work with future exploration of postuse acceptability.

Overall, the respondents were overwhelmingly positive to the idea of an app that uses GPS to track an individual’s location to facilitate connection to care. The respondents largely seemed to understand the concept and could articulate potential benefits. Few concerns with privacy were raised, and trust in researchers was noted. These results support continued development of GPS-based mHealth interventions to improve engagement in HIV care in this population.

## Appendix

Multimedia Appendix 1 Focus group discussion guide.

## Abbreviations

ART: antiretroviral therapy
FGD: focus group discussion
IDI: in-depth interview
mHealth: mobile health
NIH: National Institutes of Health
UCT: University of Cape Town
