# Supplementary material for: Attitudes Toward a Proposed GPS-Based Location Tracking Smartphone App for Improving Engagement in HIV Care Among Pregnant and Postpartum Women in South Africa: Focus Group and Interview Study
Source: JMIR Form Res. 2021 Feb 8;5(2):e19243. doi: 10.2196/19243 (PMC7899801; doi:10.2196/19243)
Supplement: Multimedia Appendix 1 [file formative_v5i2e19243_app1.docx]

**Supplemental material: Questions from focus group discussion guide**

*The application we would like to develop would help to connect women who travel to ART clinics in the new area. For example, if a woman returns to the Eastern Cape after giving birth and stays for a few months, she could open the app on her phone and look up a new ART clinic.*

1. What do you think about using a cell phone app to look up other ART clinics?
2. Would you want to do this? Why or why not?
3. What concerns would you have about receiving messages about nearby ART clinics?

*The last feature of the app would be invisible. It would use the phone’s GPS, or mapping ability, to follow the movement of the person holding the phone. We would like to do this to better understand how women travel around the time of pregnancy and how they access care. In our example, we would be able to tell that the woman has traveled to the Eastern Cape because the phone would record her general location coordinates. When I say general, I mean that we could tell the town or village she’s in, but not the specific building, because that is not of concern to us. We as researchers then would use that information to describe how women who use the app travel around and why they may not return to care in Gugulethu.*

1. What do you think about a cell phone recording location so that researchers can use it?
2. Would you want to do this? Why or why not?
3. Are there any privacy issues that concern you with the app we have described?
